# Mitotic Spindle Assembly around RCC1-Coated Beads in *Xenopus* Egg Extracts

**DOI:** 10.1371/journal.pbio.1001225

**Published:** 2011-12-27

**Authors:** David Halpin, Petr Kalab, Jay Wang, Karsten Weis, Rebecca Heald

**Affiliations:** Department of Molecular & Cell Biology, University of California–Berkeley, Berkeley, California, United States of America; University of North Carolina at Chapel Hill, United States of America

## Abstract

During cell division the genetic material on chromosomes is distributed to daughter cells by a dynamic microtubule structure called the mitotic spindle. Here we establish a reconstitution system to assess the contribution of individual chromosome proteins to mitotic spindle formation around single 10 µm diameter porous glass beads in *Xenopus* egg extracts. We find that Regulator of Chromosome Condensation 1 (RCC1), the Guanine Nucleotide Exchange Factor (GEF) for the small GTPase Ran, can induce bipolar spindle formation. Remarkably, RCC1 beads oscillate within spindles from pole to pole, a behavior that could be converted to a more typical, stable association by the addition of a kinesin together with RCC1. These results identify two activities sufficient to mimic chromatin-mediated spindle assembly, and establish a foundation for future experiments to reconstitute spindle assembly entirely from purified components.

## Abstract 2

Beads coated with the guanine nucleotide exchange factor RCC1 and a kinesin motor protein are sufficient to induce mitotic spindle assembly in *Xenopus* egg cytoplasm.

## Author Summary

The mitotic spindle is a bipolar structure that is responsible for separating the two sets of duplicated chromosomes in a dividing cell, thereby delivering one set to each of the two daughter cells. It is built from dynamic filaments called microtubules, as well as hundreds of other components that contribute to the organization and dynamics of the microtubules and to chromosome movement. To understand which proteins are essential for spindle formation and function, we would like to be able to build it from purified components. As a step towards this goal, we coupled individual proteins to inert glass beads (as a substitute for chromosomes), to determine what combination of proteins can induce spindle assembly in a complex cytoplasm derived from frog eggs. We found that a single enzyme called RCC1 is sufficient to activate a pathway that stabilizes and organizes microtubules into a bipolar structure around the bead, but that this bead then oscillated back and forth between the poles of the spindle. By coupling a microtubule-based motor protein together with RCC1 on the bead, we were able to balance the bead in the center of the spindle. Thus, two proteins immobilized on a bead can substitute for a chromosome and induce stable spindle formation.

## Introduction

The spindle is a highly dynamic structure composed of microtubule polymers and hundreds of other factors including motor proteins and microtubule-associated proteins (MAPs) [1]. Its purpose is to attach to chromosomes and accurately segregate them to daughter cells. Once thought to be passive participants, chromosomes are now known to play an active role in spindle assembly, since immobilized mitotic chromatin [2]–[4], or chromosome fragments containing microtubule attachment sites (kinetochores) [5], have been shown to direct the formation of spindle structures. However, the minimal chromosome components sufficient to generate a spindle have not been defined.

One candidate enzyme associated with chromatin that could drive spindle assembly is RCC1, the guanine nucleotide exchange factor (GEF) for the small GTPase Ran. RCC1 generates a steep gradient of RanGTP near chromosomes, activating a subset of mitotic motors and MAPs that are cargoes of the importin β family of nuclear transport receptors [6]. Addition of a hydrolysis-deficient mutant of Ran bound to GTP (RanQ69L-GTP) stabilizes microtubules that are organized by motor proteins into asters and small spindle-like structures in metaphase-arrested cytoplasmic extracts prepared from *Xenopus laevis* eggs [7]–[10]. RanQ69L-GTP disrupts the RCC1-generated RanGTP gradient and spindle assembly [11], while flattening the gradient eliminates spindle assembly around chromatin beads [12]. These experiments demonstrate that a RanGTP gradient is required for chromatin-dependent spindle assembly in *Xenopus* egg extracts, but is it sufficient? We set out to test whether immobilized RCC1 in the absence of other chromatin factors can reconstitute a mitotic spindle.

## Results and Discussion

**Figure 1:**
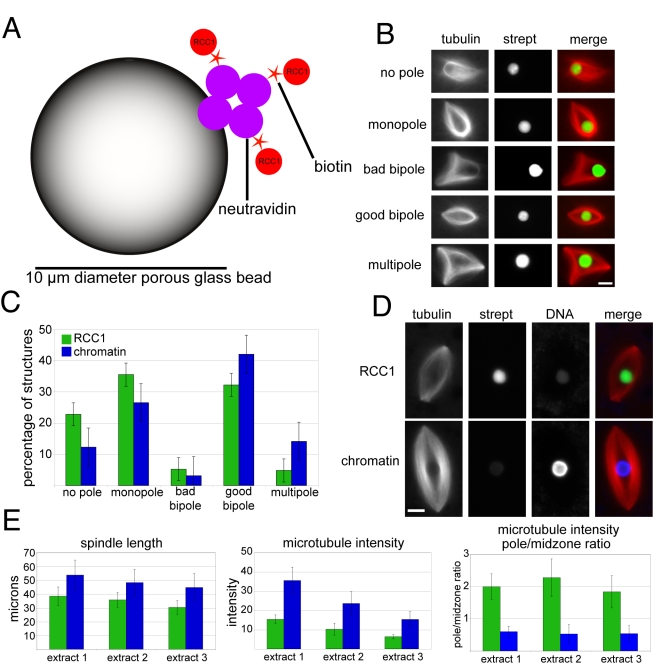
RCC1-coated beads induce spindle formation in *Xenopus laevis* egg extracts. (A) Schematic of a porous glass NeutrAvidin bead to which N-terminally biotinylated RCC1 is coupled, drawing not to scale. (B) Categories of microtubule structures formed around RCC1 beads. (C) Distribution of spindle structure categories formed around single RCC1 or chromatin beads. *N* = 3 extracts, 80–130 structures counted per extract. (D) Images comparing representative RCC1 and chromatin bead spindles. Weak signals in the DNA channel of RCC1 bead spindle and the Alexa Fluor 488-labeled Streptavidin (strept) channel of chromatin bead spindle are due to slight bead autofluorescence. (E) Quantification of differences between RCC1 and chromatin bead spindle lengths, overall spindle microtubule intensity, and pole/midzone ratio of spindle microtubule intensity. Intensity is in arbitrary units. 80–130 structures counted per extract. Microtubules are red, RCC1 beads are green, and DNA beads are blue. Scale bars, 10 µm.

To test whether a single protein factor, RCC1, is sufficient to direct spindle formation in egg extracts, we developed a novel substrate consisting of single 10 µm diameter porous NeutrAvidin beads. This approach alleviates the need for small beads to cluster or align by generating a high surface area to which biotinylated molecules can be tightly bound (Figure 1A). RCC1 (α isoform) engineered to contain a single amino-terminal biotin was coupled to the beads, which were then incubated in metaphase-arrested egg extracts containing rhodamine-labeled tubulin and observed by fluorescence microscopy. Whereas uncoupled or bovine serum albumin (BSA)-coupled NeutrAvidin beads had no activity (unpublished data), microtubule arrays formed around RCC1 beads that could be sorted into five major categories (Figure 1B). Robust bipolar structures made up greater than 30% of the arrays, with a distribution of categories similar to single chromatin-coated beads under the same reaction conditions (Figure 1C). Notably, however, RCC1 bead spindle morphology differed from that of individual chromatin beads, which induced larger spindles that contained more microtubules as determined by microtubule fluorescence intensity (Figure 1D,E). Furthermore, whereas chromatin bead spindle microtubules were most dense in the center, RCC1 bead spindle microtubules had a higher density at the poles (Figure 1D). Thus, immobilization of a single chromatin component, RCC1, is sufficient to induce bipolar spindle formation in *X. laevis* egg extracts, but spindle morphology is different, suggesting that the pathway is not completely active, or that other chromatin proteins contribute to microtubule stabilization and organization.

**Figure 2:**
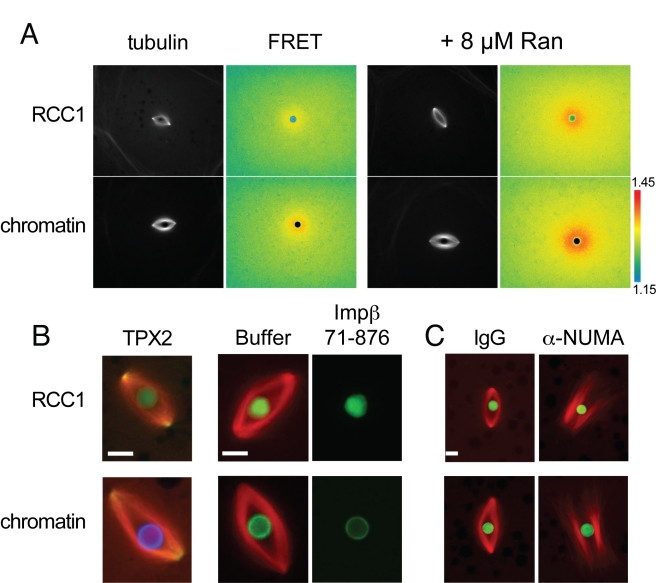
RCC1 beads fully activate the RanGTP spindle assembly pathway and share structural and dynamic features with chromatin bead spindles. (A) RanGTP induced cargo gradients visualized using the Rango FRET probe appear qualitatively similar around RCC1 and chromatin-coated beads and are enhanced by addition of exogenous Ran. Elevated FRET indicative of cargo release is red/yellow. (B) GFP-tagged importin β cargo TPX2 localizes similarly to RCC1 and chromatin bead spindles. Inhibition of cargo release by addition of importin β (71–876) similarly blocks microtubule assembly around both bead types. (C) RCC1 and chromatin bead spindles display similar sensitivity to disruption of pole formation upon addition of α-NuMA antibodies. Microtubules are red, beads are green or blue, and TPX2 is green. See Figure S2 for individual fluorescence image channels. Scale bars, 10 µm. Reactions in (B) and (C) were supplemented with 8–10 µM Ran.

To determine whether RCC1 beads fully recapitulate RanGTP-driven mitotic cargo activation, we added the FRET probe Rango-2, which measures the level of cargo release from importin β [11],[13]. Rango gradients surrounding RCC1 beads appeared similar to those formed around chromatin beads, and both were further enhanced by addition of wild-type Ran, indicating that the RCC1 beads have similar activity compared to chromatin and that cargo gradients are limited by the amount of Ran that can be loaded with GTP (Figure 2A). Consistent with this interpretation, varying the amount of RCC1 per bead 4-fold had little effect on the distribution of microtubule structures (unpublished data), whereas addition of wild-type Ran up to three times its estimated endogenous concentration of ∼3 µM resulted in a dose-dependent decrease in monopolar and bad bipolar structures, while the percentage of multipolar structures indicative of enhanced microtubule polymerization increased (Figure S1). Further evidence of equivalent Ran pathway activity by RCC1 beads compared to chromatin was the similar localization of the cargo TPX2 to spindle microtubules, and sensitivity to spindle disruption by a truncation mutant of importin β (amino acids 71–876) that still binds cargo but does not bind RanGTP (Figure 2B; Figure S2) [14],[15]. Despite their morphological differences, chromatin and RCC1 bead spindle dynamics and organization were similar. Both spindle types displayed poleward microtubule flux at similar rates, indicating kinesin-5 activity (unpublished data) [16],[17], and spindle morphology was similarly disrupted upon inhibition of the microtubule cross-linking spindle pole protein NuMA (Figure 2C; Figure S2) [18]. Therefore, RCC1 beads appear to fully activate the RanGTP cargo release pathway and generate spindles that possess structural and dynamic features of spindles formed around sperm chromosomes or chromatin-coated beads.

**Figure 3:**
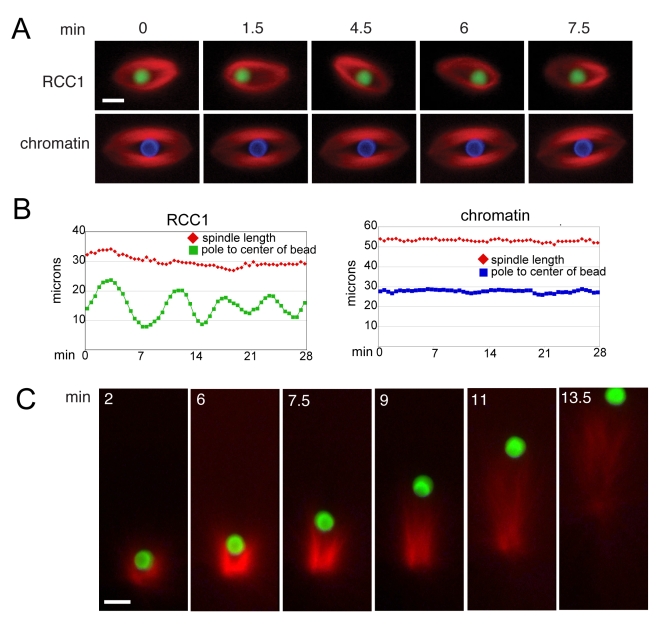
RCC1 beads oscillate pole-to-pole within the spindle and appear to be pushed by polymerizing microtubules. (A) Selected frames from Video S1 showing a stationary chromatin bead and an oscillating RCC1 bead. (B) Plots of bead position relative to one pole and spindle length over time. (C) Selected frames from Video S2 showing a forming monopolar RCC1 bead structure that pushes the bead, which appears to be trailed by polymerizing microtubules. Scale bars, 10 µm. Reactions were supplemented with 8–10 µM Ran.

One distinctive behavior observed by time-lapse fluorescence microscopy was the tendency of single RCC1 beads to oscillate within the spindle from pole to pole, whereas chromatin beads were stationary (Figure 3A,B; Video S1). We observed a range in bead oscillatory activity, which sometimes dampened over time. Interestingly, when a monopolar microtubule array formed, the RCC1 bead moved unidirectionally, appearing to be pushed along by a trail of polymerizing microtubules (Figure 3C; Video S2). This motility was reminiscent of actin polymerization-driven propulsion of the bacterium *Listeria monocytogenes* or beads coated with its actin nucleation promoting protein ActA, which does not require motor activity [19]. We therefore propose that the oscillatory movement of an RCC1 bead occurs because the bead does not connect to spindle microtubules and is pushed from pole to pole by polymerizing microtubule plus ends. Analogous to a bead uniformly coated with ActA that induces polarized actin assembly, a symmetry-breaking event might initiate RCC1 bead motility [20],[21]. Because of the antiparallel orientation of microtubules in bipolar structures, the RCC1 bead is driven towards the opposite spindle pole where it encounters a higher density of polarized microtubules polymerizing in the opposite direction. Such a polar ejection force of spindle microtubules has been well documented, although oscillatory chromosome movement also requires kinetochore fibers [22]. We cannot rule out that bead movement is regulated by egg extract factors, but biochemical association of specific extract proteins was not observed (unpublished data). We therefore reasoned that additional chromatin factors normally act to stabilize interaction with spindle microtubules.

**Figure 4:**
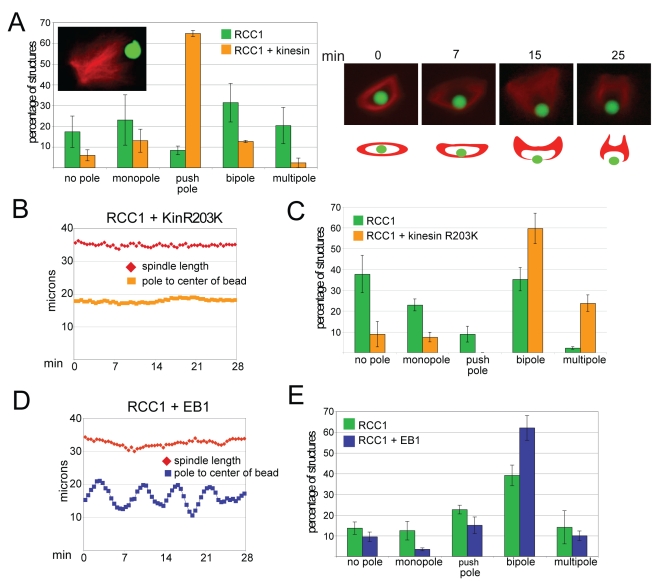
Kinesin or EB1 coupled together with RCC1 affect bead spindle dynamics and bipolarity. (A) Quantification of spindle structure categories when the conventional kinesin motor domain (kinesin-1, amino acids 1–360) is coupled to the bead together with RCC1, which predominantly yields a “push pole” phenotype (inset). *N* = 3 extracts, 80–130 structures counted per extract. Left panel shows frames from Video S3 and schematic showing how the push pole structure forms. (B) Plot of bead position and spindle length for RCC1 plus kinesin ATPase mutant R203K. See Video S4. (C) Comparison of spindle categories of RCC1 beads with or without kinesin-1 (R203K) after 1 h in egg extract shows that the kinesin ATPase mutant increases the percentage of bipolar and multipolar spindles. *N* = 3 extracts, 80–130 structures counted per extract. (D) Plot of bead position and spindle length for RCC1 plus EB1. See Video S5. (E) Comparison of spindle categories of RCC1 beads with or without EB1 after 1 h in egg extract shows that the EB1 increases the percentage of bipolar spindles. *N* = 3 extracts, 100–170 structures counted per extract. Scale bar, 10 µm. Reactions were supplemented with 8–10 µM Ran.

Plus end-directed chromatin-associated kinesins (chromokinesins) contribute to multiple aspects of mitosis, regulating spindle microtubule dynamics and organization, as well as chromosome compaction and segregation [23], and represent excellent candidate factors for mediating chromatin-spindle interactions. Furthermore, kinesin-coated beads can move directionally along microtubules in a reconstituted system [24]. To determine whether plus end-directed microtubule-based motor activity in the absence of other chromokinesin functions was sufficient to stabilize RCC1 bead spindles, we first coupled conventional kinesin-1 motor domain (amino acids 1–560) together with RCC1 to the beads at a 1∶1 ratio of proteins. Although bipolar spindles initially formed around the hybrid beads, the poles eventually collapsed together and pushed the bead out of the spindle, generating a “push pole” morphology (Figure 4A, Video S3). These observations suggest that, like around chromatin and RCC1 beads, microtubules are nucleated in random orientations and quickly attain an antiparallel organization due to the activity of kinesin-5 and other motors [4],[25]. Once microtubule plus ends become oriented toward the bead, however, the strong processive motor activity of kinesin-1 appeared to dominate microtubule organization, clustering plus ends at the surface of the bead. Interestingly, the glass bead often cracked once the poles were pushed together and outward, indicating that the kinesin motor can generate significant force against the bead (Video S3, right panel).

In contrast to kinesin-1, chromosomal kinesins -4 and -10 have slower motility and are weakly processive [26]. We therefore mutated the motor domain of kinesin-1, changing residue 203 from arginine to lysine (R203K) to reduce its ATPase activity and motility, but preserve microtubule binding [27]. Remarkably, beads coated with a 1∶1 ratio of RCC1 and kinesin-1(R203K) induced bipolar spindle assembly but were stationary like chromatin beads (Figure 4B, Video S4). Whereas no obvious effect on spindle morphology was observed, the percentage of bipolar spindles formed after 1 h increased by approximately 30% (Figure 4C). These results show that by mediating bead-microtubule interactions and centering within the spindle, a non-motile kinesin strongly enhances the stability of bipolar microtubule arrays induced by RCC1 beads.

To investigate whether other microtubule binding activities were sufficient to stabilize RCC1 bead spindles, we substituted the microtubule plus end binding protein EB1 for kinesin-1(R203K). EB1 associates with growing microtubules and functions to recruit a large set of proteins that regulate microtubule dynamics and interactions with cellular structures including kinetochores [28]. Unlike kinesin-1(R203K), the presence of EB1 on RCC1 beads did not decrease the percentage of beads oscillating or their movement amplitudes, which appeared indistinguishable compared to RCC1 beads (Figures 4D, 3B; Video S5). Nevertheless, the percentage of bipolar spindles formed at 1 h increased, similar to the RCC1/kinesin-1(R203K) hybrid beads (Figure 4C,E). EB1 may be mediating bead-spindle microtubule interactions that promote spindle bipolarity, but through a distinct microtubule binding mechanism. Alternatively, EB1 on beads may be acting directly or indirectly to stabilize spindle microtubules, thereby facilitating the activity of kinesin-5 and other motors to sort them into bipolar arrays. These results indicate that bead oscillations per se do not impair spindle bipolarity, and differential effects on the spindle likely reflect the distinct properties of EB1 and kinesin. We hypothesize that molecular motor domains provide the microtubule interaction best suited to stabilize RCC1 bead or chromosome arm position within a spindle. Evaluation of additional microtubule binding proteins or domains, and careful analysis of bead spindle phenotypes and dynamics will be necessary to elucidate underlying mechanisms.

In summary, stable bipolar spindle assembly can be induced in the absence of chromosomes, chromatin, or kinetochores by coupling two proteins, RCC1 and kinesin-1 (R203K), to beads. These results demonstrate that the anisotropy of RanGTP distribution in the cytoplasm is sufficient to drive mitotic spindle assembly. However, chromatin spindles are still larger and more robust, indicating that other chromatin-associated factors must also contribute to normal spindle morphology. One possible activity is the Aurora B kinase, which phosphorylates and inactivates microtubule-destabilizing proteins [29],[30] and makes important contributions to kinetochore-driven spindle assembly [12]. Enrichment of mitotic kinases on beads in *Xenopus* egg extracts has previously been achieved by adding beads coupled with IgGs specific for Aurora A [31], or for the chromosome passenger complex protein INCENP [12], which binds Aurora B. While neither kinase was found to be sufficient for spindle assembly, they clearly play supporting roles. Crucial also may be bona fide chromokinesins such as Xklp1, which has been shown to recruit the bundling MAP PRC1 to generate the antiparallel microtubule arrays of the central spindle [32]. Our system establishes a foundation to test the roles of other chromatin and spindle factors and forms the basis for spindle reconstitution entirely from defined components.

## Materials and Methods

Metaphase-arrested *X. laevis* egg extracts and biotinylated DNA for coupling to beads were prepared as described [4],[33]. Glass beads (GFS chemicals, #84503) were modified with silane, activated with glutaric anhydride/trimethylamine, and then linked to NeutrAvidin (Pierce), to which biotinylated proteins or plasmid DNA were coupled. Full-length human RCC1 (α isoform) containing a biotinylation tag was expressed and biotinylated in *Escherichia coli*, and purified by ion-exchange chromatography. Bacterially expressed 6x-histidine-tagged human kinesin motor domains purified by nickel and ion exchange chromatography were biotinylated in vitro at a single cysteine with biotin maleimide. Human EB1 with an N-terminal 6x-histidine-tag and biotinylation tag was expressed and biotinylated in *E. coli* and purified by nickel chromatography. Ratios of 1∶1 RCC1 to kinesin proteins, EB1, or biotinylated BSA were coupled to beads. Extract reactions containing beads and rhodamine-labeled tubulin were pre-incubated on ice and then spotted between slides and coverslips that had either been PEG-modified or treated with NaOH. After 30–60 min, reactions were viewed live by wide field fluorescence microscopy. Images and time-lapse movies were collected with a CCD camera and MicroManager software and analyzed using ImageJ. FRET images in the presence of Rango-2 were collected and processed as described [13],[14]. WT Ran (8–10 µM) was added to reactions described in Figures 2B,C, 3, and 4.

### Bead Synthesis

#### Silane coupling

All bead washes were performed twice with 2 ml solution unless stated otherwise. Beads were pelleted by spinning 2 min at 300×g. 100 mg of dried glass beads (GFS chemicals, #84503) weighed out in a 2 ml eppendorf tube were washed with acetone, then water, then 0.2 M NaOH. Beads were rotated for 30 min in 0.2 M NaOH, then washed once with water, and once with 0.2 M HCl. Beads were rotated for 30 min in 0.2 M HCl, then washed with water until neutral, then washed once more with water. Beads were then washed with ethanol and pelleted. In a separate tube, 400 µl of aminopropyl silane (Gelest #SIA0610.0) was added to 1.4 ml of 95% ethanol, 5% water, mixed, added to the beads, and rotated for 1 h. Beads were then washed 3 times with ethanol, then acetone, then blown dry with nitrogen, heated at 100°C for 30 min, and left at room temperature for 24 h to cure.

#### Glutaric anhydride coupling

The next day the beads were rinsed in dimethylformamide (DMF). 45 mg of glutaric anhydride was resuspended in 1.6 ml of DMF. The glutaric anhydride/DMF solution was mixed with the pelleted beads. 132 µl of triethylamine was added to the beads and the beads were rotated for 30 min. Beads were then washed 3 times in DMF, then water, then PBS, and resuspended in a total volume of 1 ml of PBS and stored overnight at 4°C.

#### NeutrAvidin coupling

5 mg of beads were washed twice with 200 µl of 20 mM MES pH 5.2 (MES buffer) and resuspended in 100 µl MES buffer. 20 mg of 1-(3-dimethylaminopropyl)-3-ethylcarbodiimide hydrochloride (EDC) was resuspended in 200 µl of ice-cold MES buffer, vortexed, added immediately to the beads, and rotated for 15 min at room temperature. Beads were then spun in a minitabletop centrifuge for 30 sec to pellet. Beads were washed with 300 µl of ice-cold MES buffer, pelleted, and were then resupended in 700 µl of NeutrAvidin (Pierce #31000) solution (400 µg NeutrAvidin, 140 mM sodium phosphate pH 8.0, 215 mM NaCl), and rotated at room temperature for 2 h. Beads were then washed 3 times with 200 µl of phosphate buffered saline, 0.005% Tween 20 (PBS-Tween). Beads were resuspended in 1 ml of PBS-Tween and stored at 4°C.

#### Protein binding

Biotinylated proteins were mixed together, added to 500 µg of NeutrAvidin beads, and rotated at 4°C for 30 min. Beads were then washed 3 times with 1 ml of PBS-Tween. Beads were stored in 50 µl of PBS-Tween at 4°C.

### Slide Preparation

Slides were prepared in two different ways. Microscope slides and coverslips were placed in metal racks (Electron Microscopy Sciences #72239-04 and #71420-25) rinsed thoroughly with water, then dip rinsed and stored in 10 M sodium hydroxide within a glass container. The container was then bath sonicated for 30 min. The slides and coverslips were kept within the container overnight. The following morning, slides and coverslips were rinsed with water and either (1) rinsed with ethanol, blown dry, and stored under vacuum or (2) dip rinsed in 0.2 M acetic acid for 1 min. Next they were rinsed with water. Excess water was removed by blowing with clean air, and then the racks were placed in ethanol. 1.5% hydroxy(polyethyleneoxy)propyltriethoxysilane (Gelest, #SIH6188.0) in 93.5% ethanol, 5% acetic acid was added to the slides and coverslips in a new glass containers and rocked at room temperature for 2 h. The racks were then removed from the silane solution and dip rinsed in four baths of ethanol then one bath of acetone for 1 min each. After the acetone, the slides and coverslips were blown dry with clean air and baked at 107°C for 30 min. After cooling for 1 h, they were placed in a vacuum desiccator and used for the following 2 d.

### *Xenopus laevis* Egg Extracts and Bead Assays

Approximately 10 µg (1 µl) of beads were added to 29 µl of *X. laevis* egg extracts and stored on ice with gentle tapping every 10 min. After 30–60 min, 5 µl of the reactions were spotted on a slide under a 22×22 mm coverslip, put in a humidity chamber, and kept in the dark. After 30 to 60 min on the slide, the live reactions were observed by fluorescence microscopy. Beads, microtubules, and DNA were visualized by adding 2 µg/ml Streptavidin Alexa Fluor 488 conjugate (Invitrogen #S-32354), 50 µg/ml rhodamine-labeled tubulin, and 2 µg/ml Hoechst 33342 dye, respectively, to the extract.

### Protein Expression, Labeling, and Purification

Human full-length RCC1 was cloned into pRSETA with a biotin acceptor peptide (GLNDIFEAQKIEWHE) and a spacer (ASTPPTPSPSTPPT) on the N-terminus [34]. RCC1 and the *E. coli* biotin ligase (BirA) were cotransformed into BL21 DE3 competent cells. Cells were outgrown for 1.5 h, added to 1 L of LB [35] with ampicillin and chloramphenicol, and grown overnight at 37°C. The next morning, 150 ml of culture was added to 1 L of fresh LB/amp/chlor (6 L total) and grown at 37°C until OD_600_ = 0.4. The media was brought to 50 µM biotin and protein expression induced with 0.3 mM IPTG overnight at 16°C. The next morning, cells were pelleted, then resuspended in 50 mM Tris pH 8.0, 150 mM NaCl, 4 mM EDTA, 1 mM DTT, 1 mM PMSF, 15 mM MgCl_2_, DNAse I, and lysed by French press. Lysate was filtered and run on a 5 ml SP HP column (GE Biosciences) with 20 column volumes (cv) of washes and then a gradient of 10% to 100% B for 10 cv (Buffer A: 25 mM NaPO_4_ pH 7.8, Buffer B: Buffer A+500 mM NaCl). RCC1-containing fractions were pooled and diluted down to 100 mM total salt with cold water and filtered. The protein was then loaded onto a 1 ml Q HP column (GE Biosciences) and run with the same gradient and buffers as the SP HP column. RCC1 fractions were pooled, brought to 10% glycerol, frozen in 10 µl aliquots ∼6 mg/ml in liquid nitrogen, and stored at −80°C.

Human kinesin 1–560 wild-type and kinesin 1–560 (R203K) were purified and labeled by the same protocol. Plasmids were transformed into BL21 competent cells and grown on LB plates overnight. The next morning, several colonies were combined and shaken in 20 ml of LB for 10 min. 4 ml of the bacteria was then added to 1 L of 2xYT [35] with 0.2% dextrose (4 L total) and grown at 37°C until OD_600_∼0.6. Protein expression was induced overnight at 24°C with 0.1 mM IPTG. The next morning, cells were pelleted, then resuspended in 50 mM NaHPO_4_ pH 8.0, 250 mM NaCl, 2 mM MgCl_2_, 20 mM imidazole, 1 mM ATP, 0.2 mM TCEP, 1 mM PMSF, 10 µg/ml LPC, and lysed by French press. Lysate was filtered and run on a 1 ml Nickel HiTrap column (GE Biosciences) with washes of 10 cv of buffer (50 mM NaPO_4_ pH 7.2, 250 mM NaCl, 1 mM MgCl_2_, 0.1 mM ATP, 0.2 mM TCEP) containing 20 mM imidazole followed by 20 cv with 50 mM % imidazole. Protein was eluted with 300 mM imidazole. Protein-containing fractions were pooled and diluted to 100 mM total salt with Mono Q Buffer (25 mM PIPES pH 6.8, 2 mM MgCl_2_, 1 mM EGTA, 0.1 mM ATP, 0.2 mM TCEP). The protein was then loaded onto a 1 ml Mono Q column (GE Biosciences) and eluted with a 0.1 to 1.0 M NaCl gradient over 20 cv. The highest concentration kinesin fractions were pooled and reacted on ice for 2 h with a 40 molar excess of Biotin PEG EZ link maleimide (Pierce). The reaction was then desalted over 2 HiTrap desalting columns (GE Biosciences) into 25 mM Pipes pH 6.8, 400 mM NaCl, 2 mM MgCl_2_, 1 mM EGTA, 0.1 mM ATP, 0.2 mM TCEP, 20% sucrose, aliquoted, frozen in liquid nitrogen, and stored at −80°C.

Biotinylated BSA was generated by reacting 1.25 ml of Albumin Standard (Thermo Scientific) with a 20× molar excess of EZ Link Biotin-PEO12-NHS (Thermo Scientific) on ice for 1 h. The excess biotin was removed by desalting the protein over three 5 ml HiTrap Desalting columns into PBS. Fractions were pooled, aliquoted, frozen in liquid nitrogen, and stored at −80°C.

A 6xHis-tagged human Ran construct was transformed into BL21 competent cells and grown on LB plates overnight. The next morning, several colonies were combined and shaken in 150 ml of LB for 10 min. 20 ml of the bacteria was then added to 1 L of LB (6 L total) and grown at 37°C until OD_600_∼0.4. Protein expression was induced by 0.3 mM IPTG and grown at 25°C for 4 h. Cells were pelleted and stored at −80°C. Cells were resuspended in PBS, 1 mM MgCl_2_, 0.1 mM GTP, 1 mM PMSF, and lysed with a French press. Lysate was filtered and run on a 5 ml Nickel HiTrap column (GE Biosciences) with washes of 10 cv 2% B and 20 cv 10% B. Protein was eluted with 60% B (Buffer A: 50 mM NaPO_4_ pH 7.4, 500 mM NaCl, 1 mM MgCl_2_, 0.1 mM GTP, Buffer B: Buffer A+500 mM imidazole). The highest concentration fractions were pooled and desalted over 5 HiTrap desalting columns (GE Biosciences) into XB, 1 mM MgCl_2_, 0.1 mM GTP. Desalted fractions were aliquoted, frozen in liquid nitrogen, and stored at −80°C.

Human full-length 6x-His-tagged EB1 was cloned into the pAN6 vector (Avidity), which contains a biotin acceptor peptide (GLNDIFEAQKIEWHE) on the N-terminus [34]. EB1 and the *E. coli* biotin ligase (BirA) were cotransformed into BL21 DE3 competent cells. Cells were outgrown for 1.5 h, added to 1 L of LB [35] with ampicillin and chloramphenicol, and grown overnight at 37°C. The next morning, 150 ml of culture was added to 1 L of fresh LB/amp/chlor (6 L total) and grown at 37°C until OD_600_ = 0.4. The media was brought to 50 µM biotin and protein expression induced with 0.3 mM IPTG overnight at 16°C. The next morning, cells were pelleted, then resuspended in PBS with PMSF, and lysed by French press. Lysate was filtered and run on a 5 ml Nickel HiTrap column (GE Biosciences) according to the manufacturer's instructions. Briefly, the lysate was loaded onto the column, washed with 20 cv of 100 mM imidazole, and EB1 was eluted with 300 mM imidazole. EB1-containing fractions were pooled and desalted into PBS with 3×5 ml HiTrap deslating columns (GE Biosciences). EB1 fractions were pooled and frozen in 10 µl aliquots in liquid nitrogen, and stored at −80°C.

### Bead Protein Composition

We empirically determined how much of each protein to add to the beads to obtain the desired ratios by combining different amounts of the proteins with streptavidin resin beads to couple them. After thorough washing, the bound proteins were eluted and analyzed by SDS-PAGE and their ratios determined.

## Supporting Information

Figure S1 Addition of exogenous Ran promotes spindle assembly around RCC1-coated beads. Recombinant wild-type Ran added at increasing concentrations to RCC1 bead spindle reactions causes a dose-dependent decrease in monopole and bad bipole categories, and an increase in multipolar spindles. *N* = 3 extracts, 80–130 structures counted in each experiment.
(TIF)

Figure S2 Individual fluorescence channels of merged images from Figure 2. The individual microtubule (rhodamine) or TPX2 (GFP) and bead (Alexa 488 or DNA) channels are shown as well as the merged images.
(TIF)

Video S1 Morphology and behavior of single bead spindles. Two side-by-side fluorescence time-lapse movies of egg extract spindle assembly reactions containing rhodamine labeled tubulin are shown over the same time interval of 38 min. On the left is a spindle formed around an RCC1-coated bead (green), and on the right a spindle assembled around a chromatin-coated bead (blue). Note that the RCC1 bead spindle is smaller, and that the bead oscillates from pole-to-pole (Quicktime, 1 MB).
(MOV)

Video S2 Behavior of a monopolar RCC1 bead spindle. Fluorescence time-lapse movie of an egg extract spindle assembly reaction containing rhodamine labeled tubulin is shown over a time interval of 21 min. Note that the microtubules appear to push the bead, which is trailed by microtubules (Quicktime, 1 MB).
(MOV)

Video S3 The effect of coupling kinesin 1 motor domain together with RCC1 to the bead. Two side-by-side fluorescence time-lapse movies of egg extract spindle assembly reactions containing rhodamine labeled tubulin and beads coated with RCC1 plus kinesin-1 motor domain, shown over the same time interval of 37 min. Although spindle structures form initially, the motor rapidly moves toward the microtubule plus ends, appearing to push the poles together and away from the bead, which often cracks (Quicktime, 9 MB).
(MOV)

Video S4 The effect of coupling kinesin 1 motor domain mutant R203K together with RCC1 to the bead. Two side-by-side fluorescence time-lapse movies of egg extract spindle assembly reactions containing rhodamine labeled tubulin and a bead coated either with RCC1 (left) or with RCC1 plus kinesin-1 motor domain ATPase mutant R203K, shown over the same time interval of 49 min. The non-motile kinesin stabilizes the RCC1 bead position within the center of the spindle, preventing bead oscillation (Quicktime, 1 MB).
(MOV)

Video S5 The effect of coupling EB1 together with RCC1 to the bead. Fluorescence time-lapse movie of egg extract spindle assembly reaction containing rhodamine labeled tubulin and a bead coated with RCC1 plus EB1, shown over a time interval of 40 min. EB1 does not diminish bead oscillation (Quicktime, 2.7 MB).
(MOV)

## Abbreviations

BSA: bovine serum albumin
GEF: Guanine Nucleotide Exchange Factor
MAPs: microtubule-associated proteins
RCC1: Regulator of Chromosome Condensation 1
