# Supplementary figures and images for: Mitotic Spindle Assembly around RCC1-Coated Beads in Xenopus Egg Extracts
Source: PLoS Biol. 2011 Dec 27;9(12):e1001225. doi: 10.1371/journal.pbio.1001225 (PMC3246454; doi:10.1371/journal.pbio.1001225)

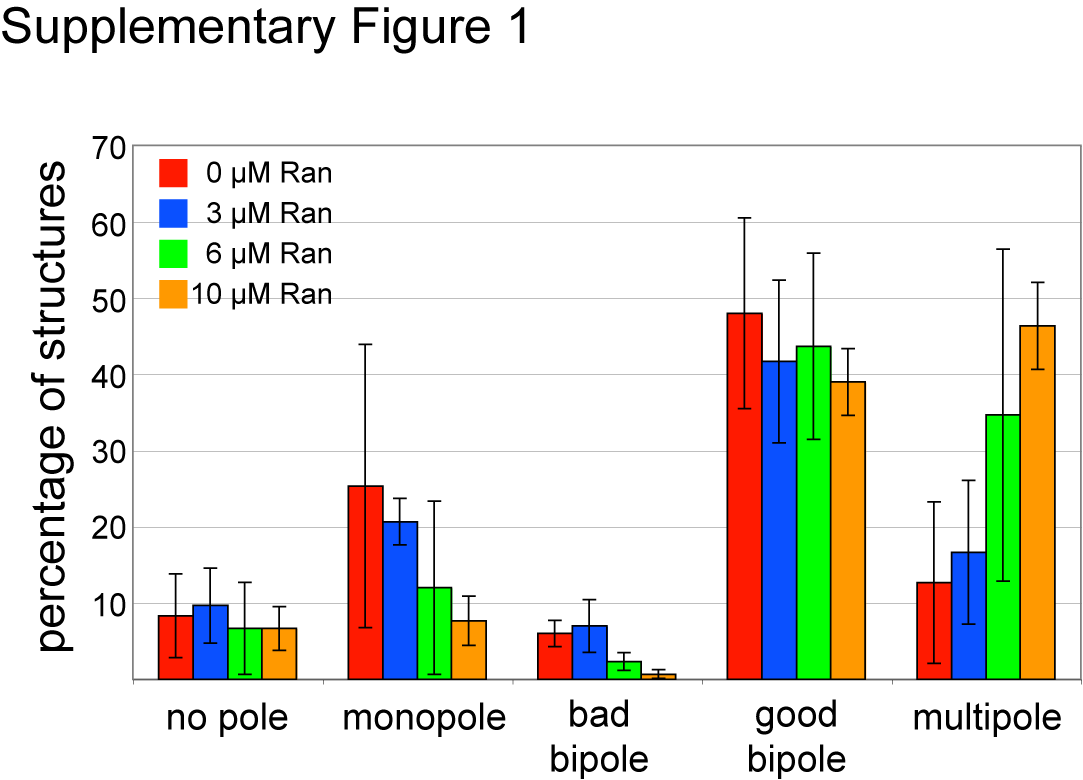

Supplement: Figure S1 — Addition of exogenous Ran promotes spindle assembly around RCC1-coated beads. Recombinant wild-type Ran added at increasing concentrations to RCC1 bead spindle reactions causes a dose-dependent decrease in monopole and bad bipole categories, and an increase in multipolar spindles. N = 3 extracts, 80–130 structures counted in each experiment. (TIF) [file pbio.1001225.s001.tif]

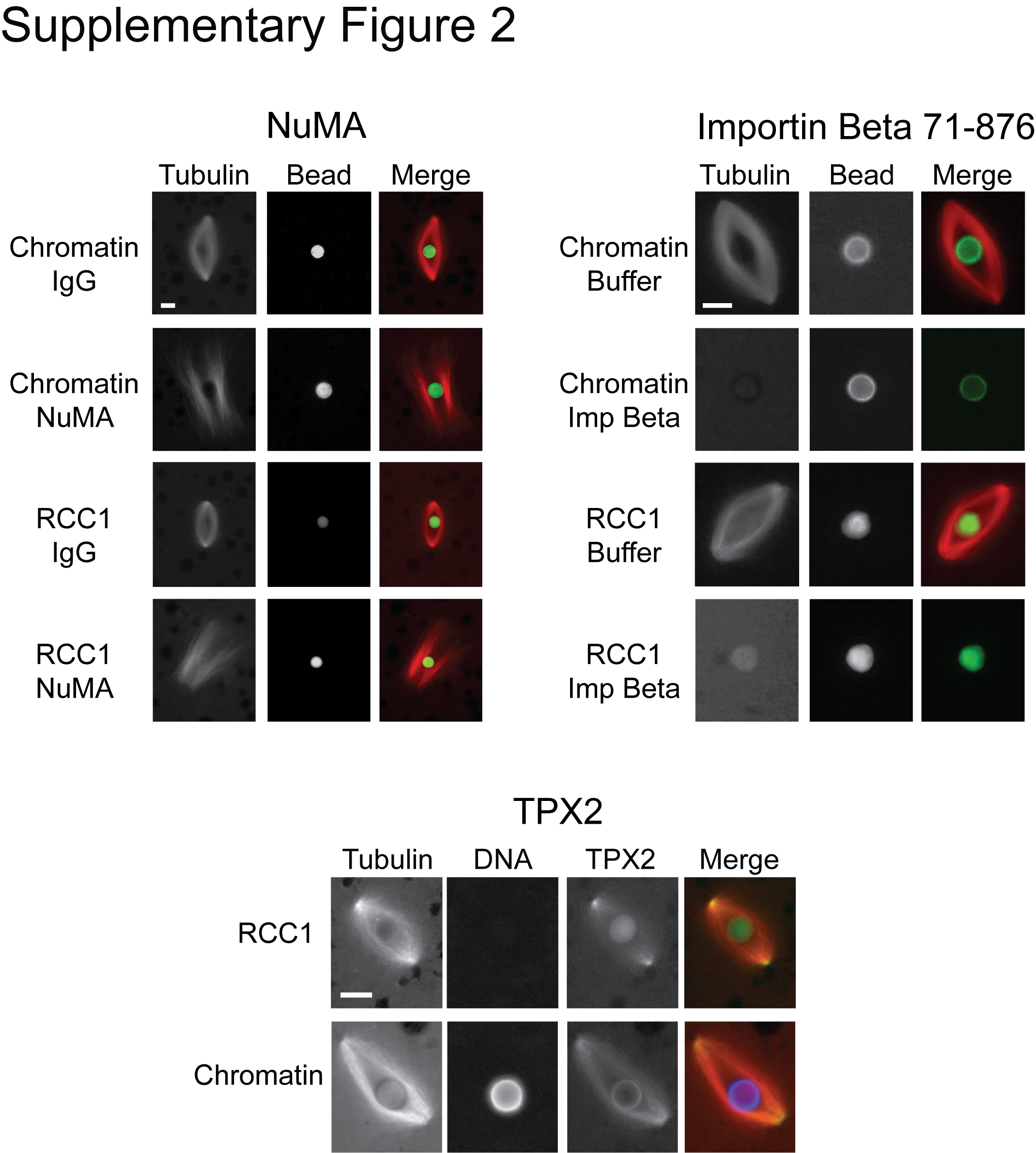

Supplement: Figure S2 — Individual fluorescence channels of merged images from Figure 2. The individual microtubule (rhodamine) or TPX2 (GFP) and bead (Alexa 488 or DNA) channels are shown as well as the merged images. (TIF) [file pbio.1001225.s002.tif]
